# Building multiclass classifiers for remote homology detection and fold recognition

**DOI:** 10.1186/1471-2105-7-455

**Published:** 2006-10-16

**Authors:** Huzefa Rangwala, George Karypis

**Affiliations:** 1 Department of Computer Science & Engineering, University of Minnesota, Minneapolis, Minnesota, USA

## Abstract

**Background:**

Protein remote homology detection and fold recognition are central problems in computational biology. Supervised learning algorithms based on support vector machines are currently one of the most effective methods for solving these problems. These methods are primarily used to solve binary classification problems and they have not been extensively used to solve the more general multiclass remote homology prediction and fold recognition problems.

**Results:**

We present a comprehensive evaluation of a number of methods for building SVM-based multiclass classification schemes in the context of the SCOP protein classification. These methods include schemes that directly build an SVM-based multiclass model, schemes that employ a second-level learning approach to combine the predictions generated by a set of binary SVM-based classifiers, and schemes that build and combine binary classifiers for various levels of the SCOP hierarchy beyond those defining the target classes.

**Conclusion:**

Analyzing the performance achieved by the different approaches on four different datasets we show that most of the proposed multiclass SVM-based classification approaches are quite effective in solving the remote homology prediction and fold recognition problems and that the schemes that use predictions from binary models constructed for ancestral categories within the SCOP hierarchy tend to not only lead to lower error rates but also reduce the number of errors in which a superfamily is assigned to an entirely different fold and a fold is predicted as being from a different SCOP class. Our results also show that the limited size of the training data makes it hard to learn complex second-level models, and that models of moderate complexity lead to consistently better results.

## Background

Breakthroughs in large-scale sequencing have led to a surge in the available protein sequence information that has far out-stripped our ability to experimentally characterize their functions. As a result, researchers are increasingly relying on computational techniques to classify proteins into functional and structural families based solely on their primary amino acid sequences. While satisfactory methods exist to detect homologs with high levels of similarity, accurately detecting homologs at low levels of sequence similarity (remote homology detection) still remains a challenging problem.

As a result, over the years several methods have been developed to address the problems of remote homology prediction and fold recognition. These include methods based on pairwise sequence comparisons [1-4], on generative models [5,6], and on discriminative classifiers [7-15].

Recent advances in string kernels that have been specifically designed for protein sequences and capture their evolutionary relationships [14,15] have resulted in the development of support vector machine-based (SVMs) [16] discriminative classifiers that show superior performance when compared to the other methods [15]. These SVM-based approaches were designed to solve *one*-*versus*-*rest *binary classification problems and to this date, they are primarily evaluated with respect to how well each binary classifier can identify the proteins that belong to its own class (e.g., superfamily or fold). However, from a biologist's perspective, the problem that he or she is facing (and would like to solve) is that of identifying the most likely superfamily or fold (or a short list of candidates) that a particular protein belongs to. This is essentially a multiclass classification problem, in which given a set of *K *classes, we would like to assign a protein sequence to one of them.

Even though highly accurate SVM-based binary classifiers can go a long way in addressing some of the biologist's requirements, it is still unknown how to best combine the predictions of a set of SVM-based binary classifiers to solve the multiclass classification problem and assign a protein sequence to a particular superfamily or fold.

Moreover, it is not clear whether schemes that combine binary classifiers are inherently better suited for solving the remote homology prediction and fold recognition problems over schemes that directly build an SVM-based multiclass classification model.

The work done by Ding *et al*. [17] recognized this problem, and used simple voting mechanism to combine the predictions obtained from binary base classifiers. They not only used, the one-versus-rest classifiers but also trained several one-versus-one classifiers, and a combination of them to obtain good classification results. The hierarchical nature, of the SCOP database was exploited by Huang *et al*. [18], such that the predictions were made in a hierarchical fashion, where a classifier was first used to classify the sequences into the four major classes, and then folds. Recently, Ie *et al*. [19], developed schemes for combining the outputs of a set of binary SVM-based classifiers for primarily solving the remote homology detection problem. Specifically borrowing ideas from error-correcting output codes [20-22], they developed schemes that use a separate learning step to learn how to best scale the outputs of the binary classifiers such that when combined with a scheme that assigns a protein to the class whose corresponding scaled binary SVM prediction is the highest, it achieves the best multiclass prediction performance. In addition, for remote homology prediction in the context of the SCOP [23] hierarchical classification scheme, they also studied the extent to which the use of such hierarchical information can further improve the performance of remote homology prediction. Their experiments showed that these approaches lead to better results than the traditional schemes that use either the maximum functional output [24] or those based on fitting a sigmoid function [25]. Finally, within the context of gene ontology classification, a Bayesian framework was recently used for combining the predictions of a hierarchy of support vector machines trained on multiple data types [26]. In this paper, motivated by the positive results of Ie *et al*'s. work [19], we further study the problem of building SVM-based multiclass classification models for remote homology detection and fold recognition in the context of the SCOP protein classification scheme. We present a comprehensive study of different approaches for building such classifiers including (i) schemes that directly build an SVM-based multiclass model, (ii) schemes that employ a second-level learner to combine the predictions generated by a set of binary SVM-based classifiers, and (iii) schemes that build and combine binary classifiers for various levels of the SCOP hierarchy. In addition, we present and study three different approaches for combining the outputs of the binary classifiers that lead to hypothesis spaces of different complexity and expressive power.

These schemes are thoroughly evaluated for both remote homology detection and fold recognition using four different datasets derived from SCOP [23]. Our experimental results show that most of the proposed multiclass SVM-based classification approaches are quite effective in solving the remote homology detection and fold recognition problems. Among them, schemes employing a two-level learning framework are in general superior to those based on the direct SVM-based multiclass classifiers, even though the performance achieved by the later schemes is quite respectable. Our results also show that the multiclass classifiers that use predictions from binary models constructed for ancestral categories within the SCOP hierarchy improve the prediction results. These schemes lead to both lower error rates and reduce the errors in which a superfamily is assigned to an entirely different fold and a fold is predicted as being from a different SCOP class. Moreover, our study shows that the limited size of the training data makes it hard to learn complex second-level models, and that models of moderate complexity lead to consistently better results.

## Results

### Algorithms For *K*-way Classification

Given a set of *m *training examples {(*x*_1_, *y*_1_),..., (*x*_*m*_, *y*_*m*_)}, where example *x*_*i *_is drawn from a domain X
 MathType@MTEF@5@5@+=feaafiart1ev1aaatCvAUfKttLearuWrP9MDH5MBPbIqV92AaeXatLxBI9gBamrtHrhAL1wy0L2yHvtyaeHbnfgDOvwBHrxAJfwnaebbnrfifHhDYfgasaacH8akY=wiFfYdH8Gipec8Eeeu0xXdbba9frFj0=OqFfea0dXdd9vqai=hGuQ8kuc9pgc9s8qqaq=dirpe0xb9q8qiLsFr0=vr0=vr0dc8meaabaqaciaacaGaaeqabaWaaeGaeaaakeaaimaacqWFxepwaaa@384F@ ⊆ ℜ^*n *^and each of the label *y*_*i *_is an integer from the set Y
 MathType@MTEF@5@5@+=feaafiart1ev1aaatCvAUfKttLearuWrP9MDH5MBPbIqV92AaeXatLxBI9gBamrtHrhAL1wy0L2yHvtyaeHbnfgDOvwBHrxAJfwnaebbnrfifHhDYfgasaacH8akY=wiFfYdH8Gipec8Eeeu0xXdbba9frFj0=OqFfea0dXdd9vqai=hGuQ8kuc9pgc9s8qqaq=dirpe0xb9q8qiLsFr0=vr0=vr0dc8meaabaqaciaacaGaaeqabaWaaeGaeaaakeaaimaacqWFyeFwaaa@3851@ = {1,..., *K*}, the goal of the *K*-way classification problem is to learn a model that assigns the correct label from the set Y
 MathType@MTEF@5@5@+=feaafiart1ev1aaatCvAUfKttLearuWrP9MDH5MBPbIqV92AaeXatLxBI9gBamrtHrhAL1wy0L2yHvtyaeHbnfgDOvwBHrxAJfwnaebbnrfifHhDYfgasaacH8akY=wiFfYdH8Gipec8Eeeu0xXdbba9frFj0=OqFfea0dXdd9vqai=hGuQ8kuc9pgc9s8qqaq=dirpe0xb9q8qiLsFr0=vr0=vr0dc8meaabaqaciaacaGaaeqabaWaaeGaeaaakeaaimaacqWFyeFwaaa@3851@ to an unseen test example. This can be thought of as learning a function *f *: X
 MathType@MTEF@5@5@+=feaafiart1ev1aaatCvAUfKttLearuWrP9MDH5MBPbIqV92AaeXatLxBI9gBamrtHrhAL1wy0L2yHvtyaeHbnfgDOvwBHrxAJfwnaebbnrfifHhDYfgasaacH8akY=wiFfYdH8Gipec8Eeeu0xXdbba9frFj0=OqFfea0dXdd9vqai=hGuQ8kuc9pgc9s8qqaq=dirpe0xb9q8qiLsFr0=vr0=vr0dc8meaabaqaciaacaGaaeqabaWaaeGaeaaakeaaimaacqWFxepwaaa@384F@ → Y
 MathType@MTEF@5@5@+=feaafiart1ev1aaatCvAUfKttLearuWrP9MDH5MBPbIqV92AaeXatLxBI9gBamrtHrhAL1wy0L2yHvtyaeHbnfgDOvwBHrxAJfwnaebbnrfifHhDYfgasaacH8akY=wiFfYdH8Gipec8Eeeu0xXdbba9frFj0=OqFfea0dXdd9vqai=hGuQ8kuc9pgc9s8qqaq=dirpe0xb9q8qiLsFr0=vr0=vr0dc8meaabaqaciaacaGaaeqabaWaaeGaeaaakeaaimaacqWFyeFwaaa@3851@ which maps each instance *x *to an element *y *of Y
 MathType@MTEF@5@5@+=feaafiart1ev1aaatCvAUfKttLearuWrP9MDH5MBPbIqV92AaeXatLxBI9gBamrtHrhAL1wy0L2yHvtyaeHbnfgDOvwBHrxAJfwnaebbnrfifHhDYfgasaacH8akY=wiFfYdH8Gipec8Eeeu0xXdbba9frFj0=OqFfea0dXdd9vqai=hGuQ8kuc9pgc9s8qqaq=dirpe0xb9q8qiLsFr0=vr0=vr0dc8meaabaqaciaacaGaaeqabaWaaeGaeaaakeaaimaacqWFyeFwaaa@3851@.

#### Direct SVM-based *K*-way Classifier Solution

One way of solving the *K*-way classification problem using support vector machines is to use one of the many multiclass formulations for SVMs that were developed over the years [27-31]. These algorithms extend the notions of separating hyperplanes and margins and learn a model that directly separates the different classes.

In this study we evaluate the effectiveness of one of these formulations that was developed by Crammer and Singer [31], which leads to reasonably efficient optimization problems.

This formulation aims to learn a matrix *W *of size *K *× *n *such that the predicted class y* for an instance *x *is given by

y*=arg⁡max⁡i=1K{〈Wi,x〉},     (1)
 MathType@MTEF@5@5@+=feaafiart1ev1aaatCvAUfKttLearuWrP9MDH5MBPbIqV92AaeXatLxBI9gBaebbnrfifHhDYfgasaacH8akY=wiFfYdH8Gipec8Eeeu0xXdbba9frFj0=OqFfea0dXdd9vqai=hGuQ8kuc9pgc9s8qqaq=dirpe0xb9q8qiLsFr0=vr0=vr0dc8meaabaqaciaacaGaaeqabaqabeGadaaakeaacqWG5bqEcqGGQaGkcqGH9aqpdaWfWaqaaiGbcggaHjabckhaYjabcEgaNjGbc2gaTjabcggaHjabcIha4bWcbaGaemyAaKMaeyypa0JaeGymaedabaGaem4saSeaaOGaei4EaSNaeyykJeUaem4vaC1aaSbaaSqaaiabdMgaPbqabaGccqGGSaalcqWG4baEcqGHQms8cqGG9bqFcqGGSaalcaWLjaGaaCzcamaabmaabaGaeGymaedacaGLOaGaayzkaaaaaa@4D50@

where *W*_*i *_is the *i*^*th *^row of *W *whose dimension is *n*.

This formulation models each class *i *by its own hyperplane (whose normal vector corresponds to the *i*^*th *^row of *W*) and assigns an example *x *to the class *i *that maximizes its corresponding hyperplane distance.

*W *itself is learned from the training data following a maximum margin with soft constraints formulation that gives rise to the following optimization problem [31]:

min⁡12βW2+∑i=1mξi,subject to:∀i,z〈Wyi,xi〉+δyi,z−〈Wz,xi〉≥1−ξi     (2)
 MathType@MTEF@5@5@+=feaafiart1ev1aaatCvAUfKttLearuWrP9MDH5MBPbIqV92AaeXatLxBI9gBaebbnrfifHhDYfgasaacH8akY=wiFfYdH8Gipec8Eeeu0xXdbba9frFj0=OqFfea0dXdd9vqai=hGuQ8kuc9pgc9s8qqaq=dirpe0xb9q8qiLsFr0=vr0=vr0dc8meaabaqaciaacaGaaeqabaqabeGadaaakeaafaqaaeGacaaabaGagiyBa0MaeiyAaKMaeiOBa4gabaWaaSaaaeaacqaIXaqmaeaacqaIYaGmaaacciGae8NSdiMaem4vaC1aaWbaaSqabeaacqaIYaGmaaGccqGHRaWkdaaeWaqaaiab=57a4naaBaaaleaacqWGPbqAaeqaaaqaaiabdMgaPjabg2da9iabigdaXaqaaiabd2gaTbqdcqGHris5aOGaeiilaWcabaGaee4CamNaeeyDauNaeeOyaiMaeeOAaOMaeeyzauMaee4yamMaeeiDaqNaeeiiaaIaeeiDaqNaee4Ba8MaeiOoaOdabaGaeyiaIiIaemyAaKMaeiilaWIaemOEaO3aaaWabeaacqWGxbWvdaWgaaWcbaGaemyEaK3aaSbaaWqaaiabdMgaPbqabaaaleqaaOGaeiilaWIaemiEaG3aaSbaaSqaaiabdMgaPbqabaaakiaawMYicaGLQmcacqGHRaWkcqWF0oazdaWgaaWcbaGaemyEaK3aaSbaaWqaaiabdMgaPbqabaWccqGGSaalcqWG6bGEaeqaaOGaeyOeI0YaaaWabeaacqWGxbWvdaWgaaWcbaGaemOEaOhabeaakiabcYcaSiabdIha4naaBaaaleaacqWGPbqAaeqaaaGccaGLPmIaayPkJaGaeyyzImRaeGymaeJaeyOeI0Iae8NVdG3aaSbaaSqaaiabdMgaPbqabaaaaOGaaCzcaiaaxMaadaqadaqaaiabikdaYaGaayjkaiaawMcaaaaa@7BDF@

where ξ_*i *_≥ 0 are slack variables, *β *> 0 is a regularization constant, and δyi,z
 MathType@MTEF@5@5@+=feaafiart1ev1aaatCvAUfKttLearuWrP9MDH5MBPbIqV92AaeXatLxBI9gBaebbnrfifHhDYfgasaacH8akY=wiFfYdH8Gipec8Eeeu0xXdbba9frFj0=OqFfea0dXdd9vqai=hGuQ8kuc9pgc9s8qqaq=dirpe0xb9q8qiLsFr0=vr0=vr0dc8meaabaqaciaacaGaaeqabaqabeGadaaakeaaiiGacqWF0oazdaWgaaWcbaGaemyEaK3aaSbaaWqaaiabdMgaPbqabaWccqGGSaalcqWG6bGEaeqaaaaa@33EF@ is equal to 1 if *z *= *y*_*i*_, and 0 otherwise. As in the binary support vector machines the dual version of the optimization problem and the resulting classifier depends only on the inner products, which allows us to use any of the recently developed protein string kernels [15].

#### Merging *K *One-vs-Rest Binary Classifiers

An alternate way of solving the *K*-way classification problem in the context of SVM is to first build a set of *K *one-versus-rest binary classification models {*f*_1_, *f*_2_,..., *f*_*K*_}, use all of them to predict an instance *x*, and then based on the predictions of these base classifiers {*f*_1_(*x*), *f*_2_(*x*),..., *f*_*K*_(*x*)} assign *x *to one of the *K *classes [20,21,25].

#### Max Classifier

A common way of combining the predictions of a set of *K *one-versus-rest binary classifiers is to assume that the *K *outputs are directly comparable and assign *x *to the class that achieved the highest one-versus-rest prediction value; that is, the prediction y* for an instance *x *is given by

y*=arg⁡max⁡i=1K{fi(x)}.     (3)
 MathType@MTEF@5@5@+=feaafiart1ev1aaatCvAUfKttLearuWrP9MDH5MBPbIqV92AaeXatLxBI9gBaebbnrfifHhDYfgasaacH8akY=wiFfYdH8Gipec8Eeeu0xXdbba9frFj0=OqFfea0dXdd9vqai=hGuQ8kuc9pgc9s8qqaq=dirpe0xb9q8qiLsFr0=vr0=vr0dc8meaabaqaciaacaGaaeqabaqabeGadaaakeaacqWG5bqEcqGGQaGkcqGH9aqpdaWfWaqaaiGbcggaHjabckhaYjabcEgaNjGbc2gaTjabcggaHjabcIha4bWcbaGaemyAaKMaeyypa0JaeGymaedabaGaem4saSeaaOGaei4EaSNaemOzay2aaSbaaSqaaiabdMgaPbqabaGccqGGOaakcqWG4baEcqGGPaqkcqGG9bqFcqGGUaGlcaWLjaGaaCzcaiabcIcaOiabiodaZiabcMcaPaaa@4AEE@

However, the assumption that the output scores of the different binary classifiers are directly comparable may not be valid, as different classes may be of different sizes and/or less separable from the rest of the dataset-indirectly affecting the nature of the binary model that was learned.

#### Cascaded SVM-Learning Approaches

A promising approach that has been explored in combining the outputs of *K *binary classification models is to formulate it as a cascaded learning problem in which a second level model is trained on the outputs of the binary classifiers to correctly solve the multiclass classification problem [19-21].

A simple model that can be learned is the scaling model in which the final prediction for an instance *x *is given by

y*=arg⁡max⁡i=1K{wifi(x)},     (4)
 MathType@MTEF@5@5@+=feaafiart1ev1aaatCvAUfKttLearuWrP9MDH5MBPbIqV92AaeXatLxBI9gBaebbnrfifHhDYfgasaacH8akY=wiFfYdH8Gipec8Eeeu0xXdbba9frFj0=OqFfea0dXdd9vqai=hGuQ8kuc9pgc9s8qqaq=dirpe0xb9q8qiLsFr0=vr0=vr0dc8meaabaqaciaacaGaaeqabaqabeGadaaakeaacqWG5bqEcqGGQaGkcqGH9aqpdaWfWaqaaiGbcggaHjabckhaYjabcEgaNjGbc2gaTjabcggaHjabcIha4bWcbaGaemyAaKMaeyypa0JaeGymaedabaGaem4saSeaaOGaei4EaSNaem4DaC3aaSbaaSqaaiabdMgaPbqabaGccqWGMbGzdaWgaaWcbaGaemyAaKgabeaakiabcIcaOiabdIha4jabcMcaPiabc2ha9jabcYcaSiaaxMaacaWLjaWaaeWaaeaacqaI0aanaiaawIcacaGLPaaaaaa@4DCB@

where *w*_*i *_is a factor used to scale the functional output of the *i*^*th *^classifier, and the set of *K w*_*i *_scaling factors make up the model that is being learned during the second level training phase [19]. We will refer to this scheme as the *scaling *scheme (S).

An extension to the above scheme is to also incorporate a shift parameter *s*_*i *_with each of the classes and learn a model whose prediction is given by

y*=arg⁡max⁡i=1K{wifi(x)+si}.     (5)
 MathType@MTEF@5@5@+=feaafiart1ev1aaatCvAUfKttLearuWrP9MDH5MBPbIqV92AaeXatLxBI9gBaebbnrfifHhDYfgasaacH8akY=wiFfYdH8Gipec8Eeeu0xXdbba9frFj0=OqFfea0dXdd9vqai=hGuQ8kuc9pgc9s8qqaq=dirpe0xb9q8qiLsFr0=vr0=vr0dc8meaabaqaciaacaGaaeqabaqabeGadaaakeaacqWG5bqEcqGGQaGkcqGH9aqpdaWfWaqaaiGbcggaHjabckhaYjabcEgaNjGbc2gaTjabcggaHjabcIha4bWcbaGaemyAaKMaeyypa0JaeGymaedabaGaem4saSeaaOGaei4EaSNaem4DaC3aaSbaaSqaaiabdMgaPbqabaGccqWGMbGzdaWgaaWcbaGaemyAaKgabeaakmaabmaabaGaemiEaGhacaGLOaGaayzkaaGaey4kaSIaem4Cam3aaSbaaSqaaiabdMgaPbqabaGccqGG9bqFcqGGUaGlcaWLjaGaaCzcamaabmaabaGaeGynaudacaGLOaGaayzkaaaaaa@518A@

The motivation behind this model is to emulate the expressive power of the z-score approach (i.e., *w*_*i *_= l/*σ*_*i*_, *s*_*i *_= -*μ*_*i*_/*σ*_*i*_) but learn these parameters using a maximum margin framework. We will refer to this as the *scale *&*shift *(SS) model.

Finally, a significantly more complex model can be learned by directly applying the Crammer-Singer multiclass formulation on the outputs of the binary classifiers. Specifically, the model corresponds to a *K *× *K *matrix *W *and the final prediction is given by

y*=arg⁡max⁡i=1K{〈Wi,f(x)〉},     (6)
 MathType@MTEF@5@5@+=feaafiart1ev1aaatCvAUfKttLearuWrP9MDH5MBPbIqV92AaeXatLxBI9gBaebbnrfifHhDYfgasaacH8akY=wiFfYdH8Gipec8Eeeu0xXdbba9frFj0=OqFfea0dXdd9vqai=hGuQ8kuc9pgc9s8qqaq=dirpe0xb9q8qiLsFr0=vr0=vr0dc8meaabaqaciaacaGaaeqabaqabeGadaaakeaacqWG5bqEcqGGQaGkcqGH9aqpdaWfWaqaaiGbcggaHjabckhaYjabcEgaNjGbc2gaTjabcggaHjabcIha4bWcbaGaemyAaKMaeyypa0JaeGymaedabaGaem4saSeaaOGaei4EaSNaeyykJeUaem4vaC1aaSbaaSqaaiabdMgaPbqabaGccqGGSaalcqWGMbGzcqGGOaakcqWG4baEcqGGPaqkcqGHQms8cqGG9bqFcqGGSaalcaWLjaGaaCzcamaabmaabaGaeGOnaydacaGLOaGaayzkaaaaaa@5061@

where *f*(*x*) = (*f*_1_(*x*), *f*_2_(*x*),..., *f*_*K*_(*x*)) is the vector containing the *K *outputs of the one-versus-rest binary classifiers. We will refer to this as the *Crammer-Singer *(CS) model.

Comparing the scaling approach to the Crammer-Singer approach we can see that the Crammer-Singer methodology is a more general version and should be able to learn a similar weight vector as the scaling approach. In the scaling approach, there is a single weight value associated with each of the classes. However, the Crammer-Singer approach has a whole weight vector of dimensions equal to the number of features per class. During the training stage, for the Crammer-Singer approach if all the weight values *w*_*i*, *j *_= 0, ∀*i *≠ *j *the weight vector will be equivalent to the scaling weight vector. Thus, we would expect the Crammer-Singer setting to fit the dataset much better during the training stage.

#### Use of Hierarchical Information

One of the key characteristics of remote homology detection and fold recognition is that the target classes are naturally organized in a hierarchical fashion. This hierarchical organization is evident in the tree-structured organization of the various known protein structures that is produced by the widely used protein structure classification schemes of SCOP [23], CATH [32] and FSSP [33].

In our study we use the SCOP classification database to define the remote homology prediction and fold recognition problems. SCOP organizes the proteins into four primary levels (class, fold, superfamily, and family) based on structure and sequence similarity. Within the SCOP classification, the problem of remote homology prediction corresponds to that of predicting the superfamily of a particular protein under the constraint that the protein is not similar to any of its descendant families, whereas the problem of fold recognition corresponds to that of predicting the fold (i.e., second level of hierarchy) under the constraint that the protein is not similar to any of its descendant superfamilies. These two constraints are important because if they are violated, then we are actually solving either the family or remote homology prediction problems, respectively.

The questions that arise are whether or not and how we can take advantage of the fact that the target classes (either superfamilies or folds) correspond to a level in a hierarchical classification scheme, so as to improve the overall classification performance?

The approach investigated in this study is primarily motivated by the different schemes presented above to combine the functional outputs of multiple one-versus-rest binary classifiers. A general way of doing this is to learn a binary one-versus-rest model for each or a subset of the nodes of the hierarchical classification scheme, and then combine these models using an approach similar to the CS-scheme.

For example, assume that we are trying to learn a fold-level multiclass model with *K*_*f *_folds where *K*_*s *_is the number of superfamilies that are descendants of these *K*_*f *_folds, and *K*_*c *_is the number of classes that are ancestors in the SCOP hierarchy. Then, we will build *K*_*f *_+ *K*_*s *_+ *K*_*c *_one-versus-rest binary classifiers for each one of the folds, superfamilies, and classes and use them to obtain a vector of *K*_*f *_+ *K*_*s *_+ *K*_*c *_predictions for a test sequence *x*. Then, using the CS approach, we can learn a second level model *W *of size *K*_*f *_× *(K*_*f *_+ *K*_*s *_+ *K*_*c*_) and use it to predict the class of *x *as

y*=arg⁡max⁡i=1K{〈Wi,f(x)〉},     (7)
 MathType@MTEF@5@5@+=feaafiart1ev1aaatCvAUfKttLearuWrP9MDH5MBPbIqV92AaeXatLxBI9gBaebbnrfifHhDYfgasaacH8akY=wiFfYdH8Gipec8Eeeu0xXdbba9frFj0=OqFfea0dXdd9vqai=hGuQ8kuc9pgc9s8qqaq=dirpe0xb9q8qiLsFr0=vr0=vr0dc8meaabaqaciaacaGaaeqabaqabeGadaaakeaacqWG5bqEcqGGQaGkcqGH9aqpdaWfWaqaaiGbcggaHjabckhaYjabcEgaNjGbc2gaTjabcggaHjabcIha4bWcbaGaemyAaKMaeyypa0JaeGymaedabaGaem4saSeaaOGaei4EaSNaeyykJeUaem4vaC1aaSbaaSqaaiabdMgaPbqabaGccqGGSaalcqWGMbGzcqGGOaakcqWG4baEcqGGPaqkcqGHQms8cqGG9bqFcqGGSaalcaWLjaGaaCzcamaabmaabaGaeG4naCdacaGLOaGaayzkaaaaaa@5063@

where *f*(*x*) is a vector of size *K*_*f *_+ *K*_*s *_+ *K*_*c *_containing the outputs of the binary classifiers.

Note that the output space of this model is still the *K*_*f *_possible folds, but the model combines information both from the fold-level binary classifiers as well as the binary classifiers for superfamily- and class-level models.

In addition to CS-type models, the hierarchical information can also be used to build simpler models by combining selective subsets of binary classifiers. In our study we experimented with such models by focusing only on the subsets of nodes that are characteristic for each target class and are uniquely determined by it. Specifically, given a target class (i.e., superfamily or fold), the path starting from that node and moving upwards towards the root of the classification hierarchy uniquely identifies a set of nodes corresponding to higher level classes containing the target class. For example, if the target class is a superfamily, this path will identify the superfamily itself, its corresponding fold, and its corresponding class in the SCOP hierarchy.

We can construct a second level classification model by combining for each target class the predictions computed by the binary classifiers corresponding to the nodes along these paths. Specifically, for the remote homology detection problem, let *K*_*s *_be the number of target superfamilies, *f*_*i*_(*x*) the prediction computed by the *i*^*th *^superfamily classifier, f∧if
 MathType@MTEF@5@5@+=feaafiart1ev1aaatCvAUfKttLearuWrP9MDH5MBPbIqV92AaeXatLxBI9gBaebbnrfifHhDYfgasaacH8akY=wiFfYdH8Gipec8Eeeu0xXdbba9frFj0=OqFfea0dXdd9vqai=hGuQ8kuc9pgc9s8qqaq=dirpe0xb9q8qiLsFr0=vr0=vr0dc8meaabaqaciaacaGaaeqabaqabeGadaaakeaacqWGMbGzdaWgaaWcbaGaey4jIK9aa0baaWqaaiabdMgaPbqaaiabdAgaMbaaaSqabaaaaa@32C4@(*x*) the prediction of the fold classifier corresponding to the *i*^*th *^superfamily, and f∧ic
 MathType@MTEF@5@5@+=feaafiart1ev1aaatCvAUfKttLearuWrP9MDH5MBPbIqV92AaeXatLxBI9gBaebbnrfifHhDYfgasaacH8akY=wiFfYdH8Gipec8Eeeu0xXdbba9frFj0=OqFfea0dXdd9vqai=hGuQ8kuc9pgc9s8qqaq=dirpe0xb9q8qiLsFr0=vr0=vr0dc8meaabaqaciaacaGaaeqabaqabeGadaaakeaacqWGMbGzdaWgaaWcbaGaey4jIK9aa0baaWqaaiabdMgaPbqaaiabdogaJbaaaSqabaaaaa@32BE@(*x*) the prediction of the class level classifier corresponding to the *i*^*th *^superfamily, then we can express the prediction for instance *x *as

y*=arg⁡max⁡i=1Kf{wifi(x)+w∧iff∧if(x)+w∧icf∧ic(x)},     (8)
 MathType@MTEF@5@5@+=feaafiart1ev1aaatCvAUfKttLearuWrP9MDH5MBPbIqV92AaeXatLxBI9gBaebbnrfifHhDYfgasaacH8akY=wiFfYdH8Gipec8Eeeu0xXdbba9frFj0=OqFfea0dXdd9vqai=hGuQ8kuc9pgc9s8qqaq=dirpe0xb9q8qiLsFr0=vr0=vr0dc8meaabaqaciaacaGaaeqabaqabeGadaaakeaacqWG5bqEcqGGQaGkcqGH9aqpdaWfWaqaaiGbcggaHjabckhaYjabcEgaNjGbc2gaTjabcggaHjabcIha4bWcbaGaemyAaKMaeyypa0JaeGymaedabaGaem4saS0aaSbaaWqaaiabdAgaMbqabaaaaOGaei4EaSNaem4DaC3aaSbaaSqaaiabdMgaPbqabaGccqWGMbGzdaWgaaWcbaGaemyAaKgabeaakmaabmaabaGaemiEaGhacaGLOaGaayzkaaGaey4kaSIaem4DaC3aaSbaaSqaaiabgEIizpaaDaaameaacqWGPbqAaeaacqWGMbGzaaaaleqaaOGaemOzay2aaSbaaSqaaiabgEIizpaaDaaameaacqWGPbqAaeaacqWGMbGzaaaaleqaaOWaaeWaaeaacqWG4baEaiaawIcacaGLPaaacqGHRaWkcqWG3bWDdaWgaaWcbaGaey4jIK9aa0baaWqaaiabdMgaPbqaaiabdogaJbaaaSqabaGccqWGMbGzdaWgaaWcbaGaey4jIK9aa0baaWqaaiabdMgaPbqaaiabdogaJbaaaSqabaGcdaqadaqaaiabdIha4bGaayjkaiaawMcaaiabc2ha9jabcYcaSiaaxMaacaWLjaWaaeWaaeaacqaI4aaoaiaawIcacaGLPaaaaaa@6FB4@

where *w*_*i*_, w∧if
 MathType@MTEF@5@5@+=feaafiart1ev1aaatCvAUfKttLearuWrP9MDH5MBPbIqV92AaeXatLxBI9gBaebbnrfifHhDYfgasaacH8akY=wiFfYdH8Gipec8Eeeu0xXdbba9frFj0=OqFfea0dXdd9vqai=hGuQ8kuc9pgc9s8qqaq=dirpe0xb9q8qiLsFr0=vr0=vr0dc8meaabaqaciaacaGaaeqabaqabeGadaaakeaacqWG3bWDdaWgaaWcbaGaey4jIK9aa0baaWqaaiabdMgaPbqaaiabdAgaMbaaaSqabaaaaa@32E6@ and  w∧ic
 MathType@MTEF@5@5@+=feaafiart1ev1aaatCvAUfKttLearuWrP9MDH5MBPbIqV92AaeXatLxBI9gBaebbnrfifHhDYfgasaacH8akY=wiFfYdH8Gipec8Eeeu0xXdbba9frFj0=OqFfea0dXdd9vqai=hGuQ8kuc9pgc9s8qqaq=dirpe0xb9q8qiLsFr0=vr0=vr0dc8meaabaqaciaacaGaaeqabaqabeGadaaakeaacaaMc8Uaem4DaC3aaSbaaSqaaiabgEIizpaaDaaameaacqWGPbqAaeaacqWGJbWyaaaaleqaaaaa@346B@ are scaling factors learned during training of the second level model.

Note that the underlying model in Equation 8 is essentially an extension of the scaling model of Equation 4 as it linearly combines the predictions of the binary classifiers of the ancestor nodes.

In a similar fashion, we can use the scale and shift type approach for every node in the hierarchical tree. This allows for an extra shift parameter to be associated with each of the nodes being modeled. Note that similar approaches can be used to define models for fold recognition, where a weight vector is learned to combine the target fold level node along with its specific class level node. A model can also be learned by not considering all the levels along the paths to the root of the tree.

The generic problem of classifying within the context of a hierarchical classification system has recently been studied by the machine learning community and a number of alternative approaches have been developed [34-36].

### Implementation

We learn the weight vector by a cross-validation set-up on the training set using either the ranking perceptron [37] or structured SVM algorithm [34] both of which work on the principles of large margin discriminative classifiers. We also introduce the notion of loss functions that are optimized for the different integration methods. The exact training methodology, including the programs used for this study are explained in the methods section.

#### Structured Output Spaces

The various models introduced for merging *K*-way one versus rest binary classifiers can be expressed using a unified framework that was recently introduced for learning in structured output spaces [34,37-39].

This framework [34] learns a discriminant function *F *: X
 MathType@MTEF@5@5@+=feaafiart1ev1aaatCvAUfKttLearuWrP9MDH5MBPbIqV92AaeXatLxBI9gBamrtHrhAL1wy0L2yHvtyaeHbnfgDOvwBHrxAJfwnaebbnrfifHhDYfgasaacH8akY=wiFfYdH8Gipec8Eeeu0xXdbba9frFj0=OqFfea0dXdd9vqai=hGuQ8kuc9pgc9s8qqaq=dirpe0xb9q8qiLsFr0=vr0=vr0dc8meaabaqaciaacaGaaeqabaWaaeGaeaaakeaaimaacqWFxepwaaa@384F@ × Y
 MathType@MTEF@5@5@+=feaafiart1ev1aaatCvAUfKttLearuWrP9MDH5MBPbIqV92AaeXatLxBI9gBamrtHrhAL1wy0L2yHvtyaeHbnfgDOvwBHrxAJfwnaebbnrfifHhDYfgasaacH8akY=wiFfYdH8Gipec8Eeeu0xXdbba9frFj0=OqFfea0dXdd9vqai=hGuQ8kuc9pgc9s8qqaq=dirpe0xb9q8qiLsFr0=vr0=vr0dc8meaabaqaciaacaGaaeqabaWaaeGaeaaakeaaimaacqWFyeFwaaa@3851@ → ℛ
 MathType@MTEF@5@5@+=feaafiart1ev1aaatCvAUfKttLearuWrP9MDH5MBPbIqV92AaeXatLxBI9gBamrtHrhAL1wy0L2yHvtyaeHbnfgDOvwBHrxAJfwnaebbnrfifHhDYfgasaacH8akY=wiFfYdH8Gipec8Eeeu0xXdbba9frFj0=OqFfea0dXdd9vqai=hGuQ8kuc9pgc9s8qqaq=dirpe0xb9q8qiLsFr0=vr0=vr0dc8meaabaqaciaacaGaaeqabaWaaeGaeaaakeaaimaacqWFBeIuaaa@377D@ over input/output pairs from which it derives predictions by maximizing *F *over the response variable for a specific given input *x*. Hence, the general form of the hypothesis *h *is

h(x;θ)=arg⁡max⁡y∈Y{F(x,y;θ)},     (9)
 MathType@MTEF@5@5@+=feaafiart1ev1aaatCvAUfKttLearuWrP9MDH5MBPbIqV92AaeXatLxBI9gBaebbnrfifHhDYfgasaacH8akY=wiFfYdH8Gipec8Eeeu0xXdbba9frFj0=OqFfea0dXdd9vqai=hGuQ8kuc9pgc9s8qqaq=dirpe0xb9q8qiLsFr0=vr0=vr0dc8meaabaqaciaacaGaaeqabaqabeGadaaakeaacqWGObaAdaqadaqaaiabdIha4jabcUda7GGaciab=H7aXbGaayjkaiaawMcaaiabg2da9maaxababaGagiyyaeMaeiOCaiNaei4zaCMagiyBa0MaeiyyaeMaeiiEaGhaleaacqWG5bqEcqGHiiIZt0uy0HwzTfgDPnwy1egaryqtHrhAL1wy0L2yHvdaiqaacqGFyeFwaeqaaOGaei4EaSNaemOray0aaeWaaeaacqWG4baEcqGGSaalcqWG5bqEcqGG7aWocqWF4oqCaiaawIcacaGLPaaacqGG9bqFcqGGSaalcaWLjaGaaCzcamaabmaabaGaeGyoaKdacaGLOaGaayzkaaaaaa@5CAF@

where *θ *denotes a parameter vector. Function *F *is a *θ*-parameterized family of functions that is designed such that *F*(*x*, *y*; *θ*) achieves the maximum value for the correct output *y*. Among the various choices for *F*, if we focus on those that are linear in a combined feature representation of inputs and outputs, *ψ*(*x*, *y*), then Equation 9 can be rewritten as [34]:

h(x;θ)=arg⁡max⁡y∈Y{〈θ,Ψ(x,y)〉}.     (10)
 MathType@MTEF@5@5@+=feaafiart1ev1aaatCvAUfKttLearuWrP9MDH5MBPbIqV92AaeXatLxBI9gBaebbnrfifHhDYfgasaacH8akY=wiFfYdH8Gipec8Eeeu0xXdbba9frFj0=OqFfea0dXdd9vqai=hGuQ8kuc9pgc9s8qqaq=dirpe0xb9q8qiLsFr0=vr0=vr0dc8meaabaqaciaacaGaaeqabaqabeGadaaakeaacqWGObaAcqGGOaakcqWG4baEcqGG7aWoiiGacqWF4oqCcqGGPaqkcqGH9aqpdaWfqaqaaiGbcggaHjabckhaYjabcEgaNjGbc2gaTjabcggaHjabcIha4bWcbaGaemyEaKNaeyicI48enfgDOvwBHrxAJfwnHbqeg0uy0HwzTfgDPnwy1aaceaGae4hgXNfabeaakiabcUha7jabgMYiHlab=H7aXjabcYcaSiabfI6azjabcIcaOiabdIha4jabcYcaSiabdMha5jabcMcaPiabgQYiXlabc2ha9jabc6caUiaaxMaacaWLjaWaaeWaaeaacqaIXaqmcqaIWaamaiaawIcacaGLPaaaaaa@61C2@

The specific form of Ψ depends on the nature of the problem and it is this flexibility that allows us to represent the hypothesis spaces introduced for merging binary models in terms of Equation 10.

For example, consider the simple scaling scheme for the problem of fold recognition (Equation 4). The input space consists of the *f*(*x*) vectors of the binary predictions and the output space Y
 MathType@MTEF@5@5@+=feaafiart1ev1aaatCvAUfKttLearuWrP9MDH5MBPbIqV92AaeXatLxBI9gBamrtHrhAL1wy0L2yHvtyaeHbnfgDOvwBHrxAJfwnaebbnrfifHhDYfgasaacH8akY=wiFfYdH8Gipec8Eeeu0xXdbba9frFj0=OqFfea0dXdd9vqai=hGuQ8kuc9pgc9s8qqaq=dirpe0xb9q8qiLsFr0=vr0=vr0dc8meaabaqaciaacaGaaeqabaWaaeGaeaaakeaaimaacqWFyeFwaaa@3851@ consists of the set of *K*_*f *_folds (labeled from 1... *K*_*f*_). Given an example *x *belonging to fold *i *(i.e., *y *= *i*), the function ψ(*x*, *y*) maps the (*x*, *y*) pair onto a *K*_*f*_-size vector whose *i*th entry (i.e., the entry corresponding to *x*'s fold) is set to *f*_*i*_(*x*) and the remaining entries are set to zero. Then, from Equation 10 we have that

h(x;θ)=arg⁡max⁡i=1Kf{〈θ,Ψ(x,i)〉}=arg⁡max⁡i=1Kf{θifi(x)},     (11)
 MathType@MTEF@5@5@+=feaafiart1ev1aaatCvAUfKttLearuWrP9MDH5MBPbIqV92AaeXatLxBI9gBaebbnrfifHhDYfgasaacH8akY=wiFfYdH8Gipec8Eeeu0xXdbba9frFj0=OqFfea0dXdd9vqai=hGuQ8kuc9pgc9s8qqaq=dirpe0xb9q8qiLsFr0=vr0=vr0dc8meaabaqaciaacaGaaeqabaqabeGadaaakeaacqWGObaAdaqadaqaaiabdIha4jabcUda7GGaciab=H7aXbGaayjkaiaawMcaaiabg2da9maaxadabaGagiyyaeMaeiOCaiNaei4zaCMagiyBa0MaeiyyaeMaeiiEaGhaleaacqWGPbqAcqGH9aqpcqaIXaqmaeaacqWGlbWsdaWgaaadbaGaemOzaygabeaaaaGccqGG7bWEcqGHPms4cqWF4oqCcqGGSaalcqqHOoqwcqGGOaakcqWG4baEcqGGSaalcqWGPbqAcqGGPaqkcqGHQms8cqGG9bqFcqGH9aqpdaWfWaqaaiGbcggaHjabckhaYjabcEgaNjGbc2gaTjabcggaHjabcIha4bWcbaGaemyAaKMaeyypa0JaeGymaedabaGaem4saS0aaSbaaWqaaiabdAgaMbqabaaaaOGaei4EaSNae8hUde3aaSbaaSqaaiabdMgaPbqabaGccqWGMbGzdaWgaaWcbaGaemyAaKgabeaakmaabmaabaGaemiEaGhacaGLOaGaayzkaaGaeiyFa0NaeiilaWIaaCzcaiaaxMaadaqadaqaaiabigdaXiabigdaXaGaayjkaiaawMcaaaaa@749E@

which is similar to Equation 4 with *θ *representing the scaling vector *w*.

Similarly, for the scale & shift approach (Equation 5), the ψ(*x*, *y*) function maps the (*x*, *y*) pair onto a feature space of size 2*K*_*f*_, where the first *K*_*f *_dimensions are used to encode the scaling factors and the second *K*_*f *_dimensions are used to encode the shift factors. Specifically, given an example *x *belonging to fold *i*, ψ(*x*, *y*) maps (*x*, *y*) onto the vector whose *i*th entry is *f*_*i*_(*x*), it's (*K*_*f *_+ *i*)^*th *^entry is one, and the remaining entries are set to zero. Then, from Equation 10 we have that

h(x;θ)=arg⁡max⁡i=1Kf{〈θ,Ψ(x,i)〉}=arg⁡max⁡i=1Kf{θifi(x)+θKf+i},     (12)
 MathType@MTEF@5@5@+=feaafiart1ev1aaatCvAUfKttLearuWrP9MDH5MBPbIqV92AaeXatLxBI9gBaebbnrfifHhDYfgasaacH8akY=wiFfYdH8Gipec8Eeeu0xXdbba9frFj0=OqFfea0dXdd9vqai=hGuQ8kuc9pgc9s8qqaq=dirpe0xb9q8qiLsFr0=vr0=vr0dc8meaabaqaciaacaGaaeqabaqabeGadaaakeaafaqaaeGacaaabaGaemiAaG2aaeWaaeaacqWG4baEcqGG7aWoiiGacqWF4oqCaiaawIcacaGLPaaaaeaacqGH9aqpdaWfWaqaaiGbcggaHjabckhaYjabcEgaNjGbc2gaTjabcggaHjabcIha4bWcbaGaemyAaKMaeyypa0JaeGymaedabaGaem4saS0aaSbaaWqaaiabdAgaMbqabaaaaOGaei4EaSNaeyykJeUae8hUdeNaeiilaWIaeuiQdKLaeiikaGIaemiEaGNaeiilaWIaemyAaKMaeiykaKIaeyOkJeVaeiyFa0habaaabaGaeyypa0ZaaCbmaeaacyGGHbqycqGGYbGCcqGGNbWzcyGGTbqBcqGGHbqycqGG4baEaSqaaiabdMgaPjabg2da9iabigdaXaqaaiabdUealnaaBaaameaacqWGMbGzaeqaaaaakiabcUha7jab=H7aXnaaBaaaleaacqWGPbqAaeqaaOGaemOzay2aaSbaaSqaaiabdMgaPbqabaGcdaqadaqaaiabdIha4bGaayjkaiaawMcaaiabgUcaRiab=H7aXnaaBaaaleaacqWGlbWsdaWgaaadbaGaemOzaygabeaaliabgUcaRiabdMgaPbqabaGccqGG9bqFcqGGSaalaaGaaCzcaiaaxMaadaqadaqaaiabigdaXiabikdaYaGaayjkaiaawMcaaaaa@7C61@

which is equivalent to Equation 5, with the first half of *θ *corresponding the scale vector *w*, and the second half corresponding to the shift vector *s*.

Finally, in the case of the Cramer-Singer approach, the ψ(*x*, *y*) function maps (*x*, *y*) onto a feature space of size *K*_*f *_× *K*_*f*_. Specifically, given a sequence *x *belonging to fold *i*, ψ(*x*, *y*) maps (*x*, *y*) onto the vector whose *K*_*f *_entries starting at (*i *- 1)*K*_*f *_are set to *f*(*x*) (i.e., the fold prediction outputs) and the remaining (*K*_*f *_- 1)*K*_*f *_entries are set to zero. Then, by rewriting Equation 10 in terms of the above combined input-output representation, we get

h(x;θ)=arg⁡max⁡i=1Kf{〈θ,Ψ(x,i)〉}=arg⁡max⁡i=1Kf{∑j=1Κfθ(i−1)Kf+jfj(x)}.     (13)
 MathType@MTEF@5@5@+=feaafiart1ev1aaatCvAUfKttLearuWrP9MDH5MBPbIqV92AaeXatLxBI9gBaebbnrfifHhDYfgasaacH8akY=wiFfYdH8Gipec8Eeeu0xXdbba9frFj0=OqFfea0dXdd9vqai=hGuQ8kuc9pgc9s8qqaq=dirpe0xb9q8qiLsFr0=vr0=vr0dc8meaabaqaciaacaGaaeqabaqabeGadaaakeaafaqaaeGacaaabaGaemiAaG2aaeWaaeaacqWG4baEcqGG7aWoiiGacqWF4oqCaiaawIcacaGLPaaaaeaacqGH9aqpdaWfWaqaaiGbcggaHjabckhaYjabcEgaNjGbc2gaTjabcggaHjabcIha4bWcbaGaemyAaKMaeyypa0JaeGymaedabaGaem4saS0aaSbaaWqaaiabdAgaMbqabaaaaOGaei4EaSNaeyykJeUae8hUdeNaeiilaWIaeuiQdKLaeiikaGIaemiEaGNaeiilaWIaemyAaKMaeiykaKIaeyOkJeVaeiyFa0habaaabaGaeyypa0ZaaCbmaeaacyGGHbqycqGGYbGCcqGGNbWzcyGGTbqBcqGGHbqycqGG4baEaSqaaiabdMgaPjabg2da9iabigdaXaqaaiabdUealnaaBaaameaacqWGMbGzaeqaaaaakmaacmqabaWaaabmaeaacqWF4oqCdaWgaaWcbaWaaeWaaeaaieGacqGFPbqAiiaacqqFsislcqqFXaqmaiaawIcacaGLPaaacqWGlbWsdaWgaaadbaGaemOzaygabeaaliabgUcaRiabdQgaQbqabaaabaGaemOAaOMaeyypa0JaeGymaedabaGae8NMdS0aaSbaaWqaaiab+zgaMbqabaaaniabggHiLdGccqWGMbGzdaWgaaWcbaGaemOAaOgabeaakmaabmaabaGaemiEaGhacaGLOaGaayzkaaaacaGL7bGaayzFaaGaeiOla4caaiaaxMaacaWLjaWaaeWaaeaacqaIXaqmcqaIZaWmaiaawIcacaGLPaaaaaa@846A@

This is equivalent to Equation 6, as *θ *can be viewed as the matrix *W *with *K*_*f *_rows and *K*_*f *_columns.

#### Ranking Perceptron

One way of learning *θ *in Equation 10, is to use the recently developed extension to Rosenblatt's linear perceptron classifier [40], called *ranking perceptron *[37]. This is an online learning algorithm that iteratively updates *θ *for each training example that is misclassified according to Equation 10. For each misclassified example *x*_*i*_, *θ *is updated by adding to it a multiple of (ψ(*x*_*i*_, *y*_*i*_) - ψ(*x*_*i*_, yi*
 MathType@MTEF@5@5@+=feaafiart1ev1aaatCvAUfKttLearuWrP9MDH5MBPbIqV92AaeXatLxBI9gBaebbnrfifHhDYfgasaacH8akY=wiFfYdH8Gipec8Eeeu0xXdbba9frFj0=OqFfea0dXdd9vqai=hGuQ8kuc9pgc9s8qqaq=dirpe0xb9q8qiLsFr0=vr0=vr0dc8meaabaqaciaacaGaaeqabaqabeGadaaakeaacqWG5bqEdaqhaaWcbaGaemyAaKgabaGamaiScQcaQaaaaaa@31C3@)), where yi*
 MathType@MTEF@5@5@+=feaafiart1ev1aaatCvAUfKttLearuWrP9MDH5MBPbIqV92AaeXatLxBI9gBaebbnrfifHhDYfgasaacH8akY=wiFfYdH8Gipec8Eeeu0xXdbba9frFj0=OqFfea0dXdd9vqai=hGuQ8kuc9pgc9s8qqaq=dirpe0xb9q8qiLsFr0=vr0=vr0dc8meaabaqaciaacaGaaeqabaqabeGadaaakeaacqWG5bqEdaqhaaWcbaGaemyAaKgabaGamaiScQcaQaaaaaa@31C3@ is given from Equation 10 (i.e., the erroneously predicted class for *x*_*i*_). This online learning framework is identical to that used in standard perceptron learning and is known to converge when the examples are linearly separable. However this convergence property does not hold when the examples are not linearly separable.

For our study, we have extended the ranking perceptron algorithm to follow a large margin classification principle whose goal is to learn *θ *that tries to satisfy the following *m *constraints (one for each of the training examples):

∀*i *〈*θ*, Ψ(*x*_*i*_, *y*_*i*_)〉 - 〈*θ*, Ψ(*x*_*i*_, yi*
 MathType@MTEF@5@5@+=feaafiart1ev1aaatCvAUfKttLearuWrP9MDH5MBPbIqV92AaeXatLxBI9gBaebbnrfifHhDYfgasaacH8akY=wiFfYdH8Gipec8Eeeu0xXdbba9frFj0=OqFfea0dXdd9vqai=hGuQ8kuc9pgc9s8qqaq=dirpe0xb9q8qiLsFr0=vr0=vr0dc8meaabaqaciaacaGaaeqabaqabeGadaaakeaacqWG5bqEdaqhaaWcbaGaemyAaKgabaGamaiScQcaQaaaaaa@31C3@)〉 ≥ *β*||*θ*||_2_,     (14)

where *y*_*i *_is *x*_*i*_'s true class and yi*=arg⁡max⁡y∈Y/yi{〈θ,Ψ(xi,y)〉〉}
 MathType@MTEF@5@5@+=feaafiart1ev1aaatCvAUfKttLearuWrP9MDH5MBPbIqV92AaeXatLxBI9gBamrtHrhAL1wy0L2yHvtyaeHbnfgDOvwBHrxAJfwnaebbnrfifHhDYfgasaacH8akY=wiFfYdH8Gipec8Eeeu0xXdbba9frFj0=OqFfea0dXdd9vqai=hGuQ8kuc9pgc9s8qqaq=dirpe0xb9q8qiLsFr0=vr0=vr0dc8meaabaqaciaacaGaaeqabaWaaeGaeaaakeaacqWG5bqEdaqhaaWcbaGaemyAaKgabaGamaiScQcaQaaakiabg2da9iGbcggaHjabckhaYjabcEgaNjGbc2gaTjabcggaHjabcIha4naaBaaaleaacqWG5bqEcqGHiiIZimaacqWFyeFwieaacqGFVaWlieGacqqF5bqEdaWgaaadbaGae0xAaKgabeaaaSqabaGccqGG7bWEcqGHPms4iiGacqaF4oqCcqGGSaalcqqHOoqwcqGGOaakcqWG4baEdaWgaaWcbaGaemyAaKgabeaakiabcYcaSiabdMha5jabcMcaPiabgQYiXlabgQYiXlabc2ha9baa@6166@. The idea behind these constraints is to force the algorithm to learn a model in which the correct predictions are well-separated from the highest scoring incorrect

**Algorithm 1 **Learning Weight Vectors with the ranking perceptron algorithm

**Input: ***m*: Number of Training Samples.

         (*x*, *y*): Training Samples.

         *β*: User constant to control separation constraints.

         *α*: Learning rate.

**Output: ***θ: *Weight Vector.

   *θ ← *0

   **while **STOPPING CRITERION = false **do**

      **for ***i *= 1 to *m ***do**

         yi*=arg⁡max⁡y∈Y〈θ,Ψ(xi,y)〉
 MathType@MTEF@5@5@+=feaafiart1ev1aaatCvAUfKttLearuWrP9MDH5MBPbIqV92AaeXatLxBI9gBamrtHrhAL1wy0L2yHvtyaeHbnfgDOvwBHrxAJfwnaebbnrfifHhDYfgasaacH8akY=wiFfYdH8Gipec8Eeeu0xXdbba9frFj0=OqFfea0dXdd9vqai=hGuQ8kuc9pgc9s8qqaq=dirpe0xb9q8qiLsFr0=vr0=vr0dc8meaabaqaciaacaGaaeqabaWaaeGaeaaakeaacqWG5bqEdaqhaaWcbaGaemyAaKgabaGamaiScQcaQaaakiabg2da9iGbcggaHjabckhaYjabcEgaNjGbc2gaTjabcggaHjabcIha4naaBaaaleaacqWG5bqEcqGHiiIZimaacqWFyeFwaeqaaOGaeyykJeocciGae4hUdeNaeiilaWIaeuiQdKLaeiikaGIaemiEaG3aaSbaaSqaaiabdMgaPbqabaGccqGGSaalcqWG5bqEcqGGPaqkcqGHQms8aaa@58A6@

         **if **yi*
 MathType@MTEF@5@5@+=feaafiart1ev1aaatCvAUfKttLearuWrP9MDH5MBPbIqV92AaeXatLxBI9gBaebbnrfifHhDYfgasaacH8akY=wiFfYdH8Gipec8Eeeu0xXdbba9frFj0=OqFfea0dXdd9vqai=hGuQ8kuc9pgc9s8qqaq=dirpe0xb9q8qiLsFr0=vr0=vr0dc8meaabaqaciaacaGaaeqabaqabeGadaaakeaacqWG5bqEdaqhaaWcbaGaemyAaKgabaGamaiScQcaQaaaaaa@31C3@ = *y*_*i *_**then**

            yi*=arg⁡max⁡y∈Y〈θ,Ψ(xi,y)〉
 MathType@MTEF@5@5@+=feaafiart1ev1aaatCvAUfKttLearuWrP9MDH5MBPbIqV92AaeXatLxBI9gBamrtHrhAL1wy0L2yHvtyaeHbnfgDOvwBHrxAJfwnaebbnrfifHhDYfgasaacH8akY=wiFfYdH8Gipec8Eeeu0xXdbba9frFj0=OqFfea0dXdd9vqai=hGuQ8kuc9pgc9s8qqaq=dirpe0xb9q8qiLsFr0=vr0=vr0dc8meaabaqaciaacaGaaeqabaWaaeGaeaaakeaacqWG5bqEdaqhaaWcbaGaemyAaKgabaGamaiScQcaQaaakiabg2da9iGbcggaHjabckhaYjabcEgaNjGbc2gaTjabcggaHjabcIha4naaBaaaleaacqWG5bqEcqGHiiIZimaacqWFyeFwaeqaaOGaeyykJeocciGae4hUdeNaeiilaWIaeuiQdKLaeiikaGIaemiEaG3aaSbaaSqaaiabdMgaPbqabaGccqGGSaalcqWG5bqEcqGGPaqkcqGHQms8aaa@58A6@

         **end if**

         **if **〈*θ*, ψ(*x*_*i*_, *y*_*i*_)〉 - 〈*θ*, ψ(*x*_*i*_, yi*
 MathType@MTEF@5@5@+=feaafiart1ev1aaatCvAUfKttLearuWrP9MDH5MBPbIqV92AaeXatLxBI9gBaebbnrfifHhDYfgasaacH8akY=wiFfYdH8Gipec8Eeeu0xXdbba9frFj0=OqFfea0dXdd9vqai=hGuQ8kuc9pgc9s8qqaq=dirpe0xb9q8qiLsFr0=vr0=vr0dc8meaabaqaciaacaGaaeqabaqabeGadaaakeaacqWG5bqEdaqhaaWcbaGaemyAaKgabaGamaiScQcaQaaaaaa@31C3@)〉 ≤ *β*||*θ*||_2 _**then**

            *θ *← *θ *+ *α*ψ(*x*_*i*_, *y*_*i*_)

            *θ *← *θ *- *α*σ(*x*_*i*_, yi*
 MathType@MTEF@5@5@+=feaafiart1ev1aaatCvAUfKttLearuWrP9MDH5MBPbIqV92AaeXatLxBI9gBaebbnrfifHhDYfgasaacH8akY=wiFfYdH8Gipec8Eeeu0xXdbba9frFj0=OqFfea0dXdd9vqai=hGuQ8kuc9pgc9s8qqaq=dirpe0xb9q8qiLsFr0=vr0=vr0dc8meaabaqaciaacaGaaeqabaqabeGadaaakeaacqWG5bqEdaqhaaWcbaGaemyAaKgabaGamaiScQcaQaaaaaa@31C3@)

         **end if**

      **end for**

   **end while**

   Return *θ*

predictions (i.e., those corresponding to yi*
 MathType@MTEF@5@5@+=feaafiart1ev1aaatCvAUfKttLearuWrP9MDH5MBPbIqV92AaeXatLxBI9gBaebbnrfifHhDYfgasaacH8akY=wiFfYdH8Gipec8Eeeu0xXdbba9frFj0=OqFfea0dXdd9vqai=hGuQ8kuc9pgc9s8qqaq=dirpe0xb9q8qiLsFr0=vr0=vr0dc8meaabaqaciaacaGaaeqabaqabeGadaaakeaacqWG5bqEdaqhaaWcbaGaemyAaKgabaGamaiScQcaQaaaaaa@31C3@). The degree of acceptable separation, which corresponds to the required margin, is given by *β*||*θ*||_2_, where *β *is a user-specified constant. Note, the margin is expressed in terms of *θ *'s length to ensure that the separation constraints are invariant to simple scaling transformations. The ranking perceptron algorithm was also used in [19]; however, that formulation used a constant margin, which was sensitive to simple scaling transformations.

Algorithm 1 shows our extended ranking perceptron algorithm that uses the constraints of Equation 14 to guide its online learning. The key steps in this algorithm are lines 8–10 that update *952 *based on the satisfaction/violation of the constraints for each one of the *m *training instances. Since the ranking perceptron algorithm is not guaranteed to converge when the examples are not linearly separable, Algorithm 1 incorporates an explicit *stopping criterion *that after each iteration it computes the training error-rate of *θ*, and terminates when *θ*'s error rate has not improved in 100 consecutive iterations. The algorithm returns the *θ *that achieved the lowest training error rate over all iterations.

#### SVM-Struct

Recently, an efficient way of learning the vector *θ *of Equation 10 has been formulated as a convex optimization problem [34]. In this approach *θ *is learned subject to the following *m *nonlinear constraints

∀i:max⁡y∈Y/yi{〈θ,Ψ(xi,y)〉}<〈θ,Ψ(xi,yi)〉.     (15)
 MathType@MTEF@5@5@+=feaafiart1ev1aaatCvAUfKttLearuWrP9MDH5MBPbIqV92AaeXatLxBI9gBamrtHrhAL1wy0L2yHvtyaeHbnfgDOvwBHrxAJfwnaebbnrfifHhDYfgasaacH8akY=wiFfYdH8Gipec8Eeeu0xXdbba9frFj0=OqFfea0dXdd9vqai=hGuQ8kuc9pgc9s8qqaq=dirpe0xb9q8qiLsFr0=vr0=vr0dc8meaabaqaciaacaGaaeqabaWaaeGaeaaakeaacqGHaiIicqWGPbqAcqGG6aGodaWfqaqaaiGbc2gaTjabcggaHjabcIha4bWcbaGaemyEaKNaeyicI4mcdaGae8hgXNfcbaGae43la8ccbiGae0xEaK3aaSbaaWqaaiab9LgaPbqabaaaleqaaOGaei4EaSNaeyykJeocciGaeWhUdeNaeiilaWIaeuiQdKLaeiikaGIaemiEaG3aaSbaaSqaaiabdMgaPbqabaGccqGGSaalcqWG5bqEcqGGPaqkcqGHQms8cqGG9bqFcqGH8aapcqGHPms4cqaF4oqCcqGGSaalcqqHOoqwcqGGOaakcqWG4baEdaWgaaWcbaGaemyAaKgabeaakiabcYcaSiabdMha5naaBaaaleaacqWGPbqAaeqaaOGaeiykaKIaeyOkJeVaeiOla4IaaCzcaiaaxMaadaqadaqaaiabigdaXiabiwda1aGaayjkaiaawMcaaaaa@6F80@

which are similar in nature to those used in the ranking perceptron algorithm (Equation 14).

The SVM-Struct [34] algorithm, is an efficient way of solving the above optimization problem in which each of the *m *nonlinear inequalities is replaced by |Y
 MathType@MTEF@5@5@+=feaafiart1ev1aaatCvAUfKttLearuWrP9MDH5MBPbIqV92AaeXatLxBI9gBamrtHrhAL1wy0L2yHvtyaeHbnfgDOvwBHrxAJfwnaebbnrfifHhDYfgasaacH8akY=wiFfYdH8Gipec8Eeeu0xXdbba9frFj0=OqFfea0dXdd9vqai=hGuQ8kuc9pgc9s8qqaq=dirpe0xb9q8qiLsFr0=vr0=vr0dc8meaabaqaciaacaGaaeqabaWaaeGaeaaakeaaimaacqWFyeFwaaa@3851@| - 1 linear inequalities resulting in a total of *m*(|Y
 MathType@MTEF@5@5@+=feaafiart1ev1aaatCvAUfKttLearuWrP9MDH5MBPbIqV92AaeXatLxBI9gBamrtHrhAL1wy0L2yHvtyaeHbnfgDOvwBHrxAJfwnaebbnrfifHhDYfgasaacH8akY=wiFfYdH8Gipec8Eeeu0xXdbba9frFj0=OqFfea0dXdd9vqai=hGuQ8kuc9pgc9s8qqaq=dirpe0xb9q8qiLsFr0=vr0=vr0dc8meaabaqaciaacaGaaeqabaWaaeGaeaaakeaaimaacqWFyeFwaaa@3851@| - 1) linear constraints and *θ *is learned using the maximum-margin principle leading to the following hard-margin problem [34]:

min⁡θsubject to 12‖θ‖22〈θ,Ψ(xi,yi)−Ψ(xi,y)〉≥1∀i,∀y∈{Y/yi}.     (16)
 MathType@MTEF@5@5@+=feaafiart1ev1aaatCvAUfKttLearuWrP9MDH5MBPbIqV92AaeXatLxBI9gBaebbnrfifHhDYfgasaacH8akY=wiFfYdH8Gipec8Eeeu0xXdbba9frFj0=OqFfea0dXdd9vqai=hGuQ8kuc9pgc9s8qqaq=dirpe0xb9q8qiLsFr0=vr0=vr0dc8meaabaqaciaacaGaaeqabaqabeGadaaakeaafaqaceWabaaabaWaaCbeaeaacyGGTbqBcqGGPbqAcqGGUbGBaSqaaGGaciab=H7aXbqabaaakeaacqqGZbWCcqqG1bqDcqqGIbGycqqGQbGAcqqGLbqzcqqGJbWycqqG0baDcqqGGaaicqqG0baDcqqGVbWBaeaaaaGaeeiiaasbaeaabmqaaaqaamaalaaabaGaeGymaedabaGaeGOmaidaamaafmaabaGae8hUdehacaGLjWUaayPcSdWaa0baaSqaaiabikdaYaqaaiabikdaYaaaaOqaamaaamaabaGae8hUdeNaeiilaWIaeuiQdK1aaeWaaeaacqWG4baEdaWgaaWcbaGaemyAaKgabeaakiabcYcaSiabdMha5naaBaaaleaacqWGPbqAaeqaaaGccaGLOaGaayzkaaGaeyOeI0IaeuiQdK1aaeWaaeaacqWG4baEdaWgaaWcbaGaemyAaKgabeaakiabcYcaSiabdMha5bGaayjkaiaawMcaaaGaayzkJiaawQYiaiabgwMiZkabigdaXaqaaiabgcGiIiabdMgaPjabcYcaSiabgcGiIiabdMha5jabgIGiolabcUha7nrtHrhAL1wy0L2yHvtyaeHbnfgDOvwBHrxAJfwnaGabaiab+Hr8zHqaaiab99caVGqaciab8Lha5naaBaaaleaacqaFPbqAaeqaaOGaeiyFa0NaeiOla4caaiaaxMaacaWLjaWaaeWaaeaacqaIXaqmcqaI2aGnaiaawIcacaGLPaaaaaa@835E@

This hard-margin problem can be converted to a soft-margin equivalent to allow errors in the training set. This is done by introducing a slack variable, *ξ*, for every nonlinear constraint of Equation 15. The soft-margin problem is expressed as [34]:

min⁡θ,ξsubjectto 12‖θ‖22+Cn∑i=1nξi,〈θ,Ψ(xi,yi)−Ψ(xi,y)〉≥1−ξi∀i,ξi≥0,∀i,∀y∈{Y/yi}.     (17)
 MathType@MTEF@5@5@+=feaafiart1ev1aaatCvAUfKttLearuWrP9MDH5MBPbIqV92AaeXatLxBI9gBaebbnrfifHhDYfgasaacH8akY=wiFfYdH8Gipec8Eeeu0xXdbba9frFj0=OqFfea0dXdd9vqai=hGuQ8kuc9pgc9s8qqaq=dirpe0xb9q8qiLsFr0=vr0=vr0dc8meaabaqaciaacaGaaeqabaqabeGadaaakeaafaqaceWabaaabaWaaCbeaeaacyGGTbqBcqGGPbqAcqGGUbGBaSqaaGGaciab=H7aXjabcYcaSiab=57a4bqabaaakeaaieaacqGFZbWCcqGF1bqDcqGFIbGycqGFQbGAcqGFLbqzcqGFJbWycqGF0baDcqGFGaaicqGF0baDcqGFVbWBaeaaaaGaeeiiaasbaeaabmqaaaqaamaalaaabaGaeGymaedabaGaeGOmaidaamaafmaabaGae8hUdehacaGLjWUaayPcSdWaa0baaSqaaiabikdaYaqaaiabikdaYaaakiabgUcaRmaalaaabaGaem4qameabaGaemOBa4gaamaaqadabaGae8NVdG3aaSbaaSqaaiabdMgaPbqabaGccqGGSaalaSqaaiabdMgaPjabg2da9iabigdaXaqaaiabd6gaUbqdcqGHris5aaGcbaWaaaWaaeaacqWF4oqCcqGGSaalcqqHOoqwdaqadaqaaiabdIha4naaBaaaleaacqWGPbqAaeqaaOGaeiilaWIaemyEaK3aaSbaaSqaaiabdMgaPbqabaaakiaawIcacaGLPaaacqGHsislcqqHOoqwdaqadaqaaiabdIha4naaBaaaleaacqWGPbqAaeqaaOGaeiilaWIaemyEaKhacaGLOaGaayzkaaaacaGLPmIaayPkJaGaeyyzImRaeGymaeJaeyOeI0Iae8NVdG3aaSbaaSqaaiabdMgaPbqabaaakeaacqGHaiIicqWGPbqAcqGGSaalcqWF+oaEdaWgaaWcbaGaemyAaKgabeaakiabgwMiZkabicdaWiabcYcaSiabgcGiIiabdMgaPjabcYcaSiabgcGiIiabdMha5jabgIGiolabcUha7nrtHrhAL1wy0L2yHvtyaeHbnfgDOvwBHrxAJfwnaGabaiab9Hr8zjab+9caVGqaciab8Lha5naaBaaaleaacqaFPbqAaeqaaOGaeiyFa0NaeiOla4caaiaaxMaacaWLjaWaaeWaaeaacqaIXaqmcqaI3aWnaiaawIcacaGLPaaaaaa@A261@

The results of classification depend on the value *C *which is the misclassification cost that determines the trade-off between the generalization capability of the model being learned and maximizing the margin. It needs to be optimized to prevent under-fitting and over-fitting the data during the training phase. Note that the SVM-Struct algorithm was also used in [19].

#### Loss Functions

The loss function plays a key role while learning *θ*, in both the SVM-struct and ranking perceptron optimizations. Till now, our discussion focused on zero-one loss that assigns a penalty of one for a misclassification and zero for a correct prediction. However, in cases where the class sizes vary significantly across the different folds, such a zero-one loss function may not be the most appropriate as it may lead to models where the rare class instances are often mispredicted. For this reason, an alternate loss function is used, in which penalty for a misclassification is inversely proportional to the class size. This implies that the misclassification of examples belonging to smaller classes weigh higher in terms of the loss. This loss function is referred to as the balanced loss [19]. For the ranking perceptron algorithm (Algorithm 1) the update rules (statements 7 and 8) need to be scaled by the loss function. In case of the SVM-Struct formulation, the balanced loss can be optimized by reweighting the definition of separation which can be done indirectly by rescaling the slack variables *ξ*_*i *_in the constraint inequalities (Equation 17).

While using the hierarchical information in the cascaded learning approaches we experimented with a *weighted *loss function where a larger penalty was assigned when the predicted label did not share the same ancestor compared to the case when the predicted and true class labels shared the same ancestors. This variation did not result in an improvement compared to the zero-one and balanced loss. Hence, we do not report results of using such hierarchical loss functions here.

### Evaluation

The performance of various schemes in terms of zero-one and balanced error is summarized in Tables 2 and 3 for remote homology detection and in Tables 4 and 5 for fold recognition. Note, the results in Tables 2 and 4 are obtained by optimizing the balanced loss function and the results in Tables 3 and 5 are obtained by optimizing the zero-one loss function. We use four datasets-sf95 and sf40 for remote homology detection, and fd25 and fd40 for fold recognition, described in detail in the methods section. We use the standard zero-one and balanced error rates for performance assessment (described in the methods section). The schemes that are included in these tables are the following: (i) the MaxClassifier, (ii) the direct *K*-way classifier, (iii) the two-level learning approaches based on either the superfamily- or fold-level binary classifiers, and (iv) the two-level learning approaches that also incorporate hierarchical information. For all two-level learning approaches (with and without hierarchical information) these tables show the results obtained by using the scaling (S), scale & shift (SS), and Crammer-Singer (CS) schemes to construct the second-level classifiers.

**Table 2:** Zero-one and Balanced error rates for the remote homology detection problem optimized for the balanced loss function.

		sf95		sf40	
		ZE	BE	ZE	BE
MaxClassifier		14.7	30.0	21.0	29.7

Direct *K*-way Classifiers		11.5	23.1	10.9	13.0
Two-Level Approaches Without Hierarchy Information					
	Scaling	9.3	16.1	10.9	13.9
Ranking Perceptron	Scale & Shift	10.1	19.5	12.1	15.8
	Crammer Singer	14.7	28.9	17.6	24.1

	Scaling	9.0	15.9	11.8	15.7
SVM-Struct	Scale & Shift	10.7	19.9	12.1	15.1
	Crammer Singer	11.6	19.4	13.0	16.3

Two-Level Approaches With Fold-level Nodes					
	Scaling	11.2	19.6	14.7	21.4
SVM-Struct	Scale & Shift	10.1	19.3	12.1	16.9
	Crammer Singer	14.7	26.0	13.0	18.2

Two-Level Approaches With Class-level and Fold-level Nodes					
	Scaling	11.2	20.2	13.0	18.8
SVM-Struct	Scale & Shift	13.5	24.7	12.1	16.8
	Crammer Singer	14.7	26.1	13.0	17.5

ZE and BE denote the zero-one error and balanced error percent rates respectively. The results were obtained by optimizing the balanced loss function.

**Table 3:** Zero-one and Balanced error rates for the remote homology detection problem optimized for the zero-one loss function.

		sf95		sf40	
		ZE	BE	ZE	BE
MaxClassifier		14.7	30.0	21.0	29.7

Direct *K*-way Classifiers		13.5	24.8	20.5	26.5

Two-Level Approaches Without Hierarchy Information					
	Scaling	10.6	18.0	11.7	16.5
Ranking Perceptron	Scale & Shift	13.2	24.5	10.9	13.4
	Crammer Singer	17.0	34.3	14.2	19.4

	Scaling	10.7	18.1	13.4	17.3
SVM-Struct	Scale & Shift	12.4	23.7	13.4	17.3
	Crammer Singer	12.7	25.2	15.5	19.8

Two-Level Approaches With Fold-level Nodes					
	Scaling	10.4	18.7	14.7	20.0
SVM-Struct	Scale & Shift	12.4	23.7	14.7	21.4
	Crammer Singer	13.8	25.0	14.7	19.6

Two-Level Approaches With Class-level and Fold-level Nodes					
	Scaling	10.9	19.1	12.6	17.7
SVM-Struct	Scale & Shift	11.2	20.9	13.4	17.8
	Crammer Singer	14.1	27.6	12.6	17.1

ZE and BE denote the zero-one error and balanced error percent rates respectively. The results were obtained by optimizing the zero-one loss function.

**Table 4:** Zero-one and Balanced error rates for the fold recognition problem optimized for the balanced loss function.

		fd25		fd40	
		ZE	BE	ZE	BE
MaxClassifier		42.0	60.3	44.4	64.6

Direct *K*-way Classifiers		38.4	52.3	40.4	56.9

	Scaling	39.5	48.7	32.5	48.0
Ranking Perceptron	Scale & Shift	38.8	51.0	29.0	43.0
	Crammer Singer	37.7	49.6	36.0	49.6

	Scaling	39.9	52.7	30.8	46.6
SVM-Struct	Scale & Shift	39.9	52.5	28.1	42.8
	Crammer Singer	41.3	50.5	31.1	43.3

Two-Level Approaches With Class-level Nodes					
	Scaling	39.2	52.4	29.9	45.0
SVM-Struct	Scale & Shift	38.1	51.6	29.0	41.7
	Crammer Singer	41.7	50.9	29.9	41.7

Two-Level Approaches With Superfamily-level Nodes					
	Scaling	40.2	52.6	30.5	44.5
SVM-Struct	Scale & Shift	40.6	52.7	29.3	42.8
	Crammer Singer	38.8	48.8	31.0	44.9

Two-Level Approaches With Superfamily-level and Class-level Nodes					
	Scaling	41.0	50.9	33.7	44.6
SVM-Struct	Scale & Shift	39.5	51.5	29.3	42.3
	Crammer Singer	40.2	51.9	30.2	42.4

ZE and BE denote the zero-one error and balanced error percent rates respectively. The results were obtained by optimizing the balanced loss function.

**Table 5:** Zero-one and Balanced error rates for the fold recognition problem optimized for the zero-one loss function.

		fd25		fd40	
		ZE	BE	ZE	BE
MaxClassifier		42.0	60.3	44.4	64.6

Direct *K*-way Classifiers		42.8	59.4	43.0	62.7

Two-Level Approaches Without Hierarchy Information					
	Scaling	39.9	52.9	32.2	50.6
Ranking Perceptron	Scale & Shift	38.4	51.3	27.3	44.8
	Crammer Singer	34.8	48.9	37.7	56.6

	Scaling	41.3	55.2	33.7	50.0
SVM-Struct	Scale & Shift	41.0	54.3	29.0	46.2
	Crammer Singer	36.6	49.4	32.5	49.6

Two-Level Approaches With Class-level Nodes					
	Scaling	39.9	52.2	31.9	50.2
SVM-Struct	Scale & Shift	38.4	52.9	29.3	44.6
	Crammer Singer	39.2	51.8	32.8	52.9

Two-Level Approaches With Superfamily-level Nodes					
	Scaling	39.5	53.9	31.3	48.8
SVM-Struct	Scale & Shift	39.9	53.4	31.3	48.4
	Crammer Singer	37.7	52.1	33.4	51.0

Two-Level Approaches With Superfamily-level and Class-level Nodes					
	Scaling	39.2	52.2	27.3	41.0
SVM-Struct	Scale & Shift	39.9	53.9	28.4	44.1
	Crammer Singer	38.8	54.7	31.3	48.0

ZE and BE denote the zero-one error and balanced error percent rates respectively. The results were obtained by optimizing the zero-one loss function.

These tables also show the performance achieved by incorporating different types of hierarchical information in the two-level learning framework. For remote homology prediction they present results that combine information from the ancestor nodes (fold and fold+class), whereas for fold recognition they present results that combine information from ancestor nodes (class), descendant nodes (superfamily), and their combination (superfamily+class).

#### Zero-one Versus Balanced Loss Function

The direct *K*-way and the two-level learning approaches can be trained using either the zero-one or the balanced loss functions (the MaxClassifier scheme does not explicitly optimize a loss function). The zero-one loss function achieved consistently worse results than those achieved by the balanced loss function for both the remote homology detection (comparing Tables 2 and 3) and the fold recognition problem (comparing Tables 4 and 5). On the average, the zero-one and balanced error rates of the zero-one loss was 10% and 20% higher than balanced loss, respectively. For this reason, our evaluation of the various schemes focuses only on the results obtained by optimizing the balanced loss function (shown in Tables 2 and 4).

#### Performance of Direct *K*-way Classifier

Comparing the direct *K*-way classifiers against the MaxClassifier approach we see that the error rates achieved by the direct approach are smaller for both the remote homology detection and fold recognition problems. In many cases these improvements are substantial. For example, the direct *K*-way classifier achieves a 10.9% zero-one error rate for sf40 compared to a corresponding error rate of 21.0% achieved by MaxClassifier. In addition, unlike the common belief that learning SVM-based direct multiclass classifiers is computationally very expensive, we found that the Crammer-Singer formulation that we used, required time that is comparable to that required for building the various binary classifiers used by the MaxClassifier approach.

#### Non-Hierarchical Two-Level Learning Approaches

Analyzing the performance of the various two-level classifiers that do not use hierarchical information we see that the scaling (S) and scale & shift (SS) schemes achieve better error rates than those achieved by the Crammer-Singer (CS) scheme. Since the hypothesis space of the CS scheme is a superset of the hypothesis spaces of the S and SS schemes, we found this result to be surprising at first. However, in analyzing the characteristics of the models that were learned we noticed that the reason for this performance difference is the fact that the CS scheme tended to overfit the data. This was evident by the fact that the CS scheme had lower error rates on the training set than either the S or SS schemes (results not reported here). Since CS's linear model has more parameters than the other two schemes, due to the fact that the size of the training set for all three of them is the same and rather limited, such overfitting can easily occur. Note that these observations regarding these three approaches hold for the two-level approaches that use hierarchical information as well. Comparing the performance of the S and SS schemes against that of the direct *K*-way classifier we see that the two-level schemes are somewhat worse for sf40 and fd25 and considerably better for sf95 and fd40. In addition, they are consistently and substantially better than the MaxClassifer approach across all four datasets.

#### SVM-Struct versus Ranking Perceptron

For the two-level approaches that do not use hierarchical information, Tables 2 and 4 show the error-rates achieved by both the ranking perceptron and the SVM-struct algorithms. From these results we can see that for the S and SS schemes, the performance achieved by the ranking perceptron are comparable to and in some cases slightly better than those achieved by the SVM-struct algorithm. However, in the case of the CS scheme, SVM-struct is superior to the perceptron and achieves substantially smaller error rates.

This relative performance of the perceptron algorithm is both surprising as well as expected. The surprising aspect is that it is able to keep up with the considerably more sophisticated, mathematically rigorous, and computationally expensive optimizers used in SVM-struct, which lend to converge to a local minimum solution that is close the global minimum. However, this behavior, especially when the results of the CS scheme are token into account, was expected because the hypothesis spaces of the S and SS schemes are rather small (the number of variables in the S and SS models are *K *and 2*K*, respectively) and as such the optimization problem is relatively easy. However, in the case of the CS scheme which is parameterized by *K*^2 ^variables, the optimization problem becomes harder, and SVM-struct's optimization framework is capable of finding a better solution. Due to this observation we did not pursue the ranking perceptron algorithm any further when we considered two-level models that incorporate hierarchy information.

#### Hierarchical Two-Level Learning Approaches

The results for remote homology prediction show that the use of hierarchical information does not improve the overall error rates. The situation is different for fold recognition in which the use of hierarchical information leads to some improvements for fd40, especially in terms of balanced error (Table 4). Also, these results show that adding information from ancestor nodes is in general better than adding information from descendant nodes, and combining both types of information can sometimes improve the classification performance.

Even though the use of hierarchical information does not improve the overall classification accuracy, as the results in Tables 6 and 7 show, it does reduce the severity of the misclassifications. Comparing the top_1 _and top_3 _error rates for the two sets of schemes, we see that by incorporating hierarchical information they achieve consistently lower error rates. For remote homology detection, there is more than 50% reduction in the error rate due to the addition of fold-and class-level information, whereas somewhat smaller gains (4%–20%) are obtained for fold recognition by incorporating class-level information. It is also interesting to note, that there is no reduction in error rates by addition of descendant node information i.e superfamily-level, in case of fold recognition problem.

**Table 6:** Error rates (top_1_, top>_3_) for the remote homology detection problem.

		sf95		sf40	
		top_1_	top_3_	top_1_	top_3_
Two-Level Approaches Without Hierarchy Information					
	Scaling	7.5	2.6	10.1	3.8
SVM-Struct	Scale & Shift	9.0	2.0	10.1	3.4
	Crammer Singer	8.1	1.7	9.2	2.5

Two-Level Approaches With Fold-level Nodes					
	Scaling	4.6	0.9	6.3	1.7
SVM-Struct	Scale & Shift	4.0	0.9	5.0	1.7
	Crammer Singer	6.6	2.6	5.0	1.7

Two-Level Approaches With Fold-level and Class-Level Nodes					
	Scaling	5.2	1.7	5.5	1.7
SVM-Struct	Scale & Shift	5.8	2.3	4.2	2.1
	Crammer Singer	6.6	2.0	5.0	1.7

The results shown in the table are optimized for the balanced loss function.

**Table 7:** Error rates (top_1_, top_3_) for the fold recognition problem.

		fd25		fd40	
		top_1_	top_3_	top_1_	top_3_
Two-Level Approaches Without Hierarchy Information					
	Scaling	38.5	24.5	25.6	15.4
SVM-Struct	Scale & Shift	37.4	24.8	24.7	15.1
	Crammer Singer	36.3	22.7	25.0	13.4
Two-Level Approaches With Class-level Nodes					
	Scaling	36.7	21.9	20.6	11.9
SVM-Struct	Scale & Shift	36.3	21.6	21.2	12.2
	Crammer Singer	37.1	22.3	25.3	13.4
Two-Level Approaches With Superfamily-level Nodes					
	Scaling	39.9	24.5	27.9	19.5
SVM-Struct	Scale & Shift	39.6	23.4	25.3	16.0
	Crammer Singer	40.6	27.3	26.7	15.1
Two-Level Approaches With Superfamily-level and Class-level Nodes					
	Scaling	39.2	25.2	20.6	13.7
SVM-Struct	Scale & Shift	38.5	23.0	20.9	12.2
	Crammer Singer	37.1	23.7	24.1	12.5

The results shown in the table are optimized for the balanced loss function.

#### Comparison with Earlier Results

As discussed in the introduction, our research in this paper was motivated by the recent work of Ie *et. al*. [19] in which they looked at the same problem of solving the *K*-way classification problem in the context of remote homology and fold recognition and presented a two-level learning approach based on the simple scaling model (S) with and without hierarchical information. Table 8 shows the results reported in that work on the common dataset and classification problems (remote homology prediction for sf95). In addition, Table 8 shows the results obtained by our algorithms using the simple scaling model and the best results achieved among the three different models that were considered in this work (i.e., S, SS, and CS).

**Table 8:** Comparative results for the remote homology detection problem on dataset sf95.

	Ie *et al *[19]		Scaling Model		Best Model	
	ZE	BE	ZE	BE	ZE	BE
Without Hierarchy Information						
Ranking Perceptron	21.8	36.7	9.3	16.1	9.3	16.1
SVM-Struct	20.7	37.6	9.0	15.9	9.0	15.9

With Fold-level Nodes						
SVM-Struct	20.4	37.5	11.2	19.6	10.1	19.3

The results for Ie *et al *were obtained from the supplementary website for the work [19], and represent the results obtained using the simple scaling model in their implementation. The results labeled "Scaling Model" correspond to the performance achieved by our two-level classifiers using the simple scaling model, whereas the results labeled "Best Model" correspond to the best performance achieved among the simple scaling, scaling & shift, and Cramer-Singer models. Both of these results were obtained from Table 2. All results were obtained by optimizing the balanced loss function. ZE and BE denote the zero-one error and balanced error percent rates respectively.

These results show that the zero-one and balanced error rates of our algorithms are in most cases less than half of that achieved by the previous algorithms. This performance advantage can be attributed to (i) differences in the one-vs-rest binary classifiers ([19] used the profile kernel [14] whereas our schemes used the SW-PSSM kernel), (ii) our implementation of the ranking perceptron allows for a better specification of the classification margin, and (iii) our results have been optimized by performing a model selection step, described in detail in the methods section.

## Discussion

The work described in this paper helps to answer three fundamental questions. First, whether or not SVM-based approaches that directly learn multiclass classification models can effectively and computationally efficiently solve the problems of remote homology prediction and fold recognition. Second, whether or not the recently developed highly accurate binary SVM-based one-vs-rest classifiers for remote homology prediction and fold recognition can be utilized to build an equally effective multiclass prediction scheme. Third, whether or not the incorporation of binary SVM-based prediction models for coarser and/or finer levels of a typical protein structure hierarchical classification scheme can be used to improve the multiclass classification performance.

The experimental evaluation of a number of previously developed methods and methods introduced in the course of this work show that, to a large extent, the answer to all three of these questions to be yes. The Crammer-Singer-based direct *K*-way classifier is able to learn effective models in a reasonable amount of time. Its classification performance is better than that of MaxClassifier and comparable to that achieved by the two-level learning schemes in three out of the four datasets. The two-level learning framework show the best overall results, producing consistently the lowest error rates. The performance of this framework greatly depends on the complexity of the hypothesis space used during the second-level learning. Complex hypothesis spaces (e.g., the one based on Crammer-Singer) tends to overfit the training dataset, whereas simpler spaces (e.g., scaling and scale & shift) produced better and more consistent results. We believe that this is a direct consequence of the limited training set size. However, since the size of the training set depends on the number of proteins with known 3D structure, this limitation is not expected to be removed in the near future. The use of hierarchical information further improves the performance of the two-level learning framework. Not only it achieves somewhat lower zero-one and balanced error rates but it also leads to a significant reduction in the number of prediction errors in which a test instance is assigned to a superfamily or fold that belongs to an entirely different fold or SCOP class from itself. As a result, in the context of protein structure prediction via comparative modeling [41], we expect that structures built from the predictions of hierarchical two-level classifiers will lead to better models.

## Conclusion

In this paper we presented various SVM-based algorithms for solving the *k*-way classification problem in the context of remote homology prediction and fold recognition. Our results show that direct *k*-way SVM-based formulations and algorithms based on the two-level learning paradigm are quite effective for solving these problems and achieve better results than those obtained by using a set of binary one-vs-rest SVM-based classifiers. Moreover, our results and analysis showed that the two-level schemes that incorporate predictions from binary models constructed for ancestral categories within the SCOP hierarchy tend to not only lead to lower error rates but also reduce the number of errors in which a superfamily is assigned to an entirely different fold and a fold is predicted as being from a different SCOP class.

## Methods

### Dataset Description

We evaluated the performance of the various schemes on four datasets. The first dataset, referred to as sf95 (superfamily – 95%), was created by Ie *et al*. [19] to evaluate the performance of the multiclass classification algorithms that they developed (sf95 was designed by Ie *et al*. [19], whereas the other three datasets, referred to as sf40 (superfamily – 40%), fd25 (fold – 25%), and fd40 (fold – 40%), were created for this study. sf40, fd25, and fd40 are available at the supplementary website.)

The sf95 dataset was derived from SCOP 1.65, whereas the other datasets were derived from SCOP 1.67. Table 1 summarizes the characteristics of these datasets and presents various sequence similarity statistics.

**Table 1:** Dataset Statistics.

Statistic	sf95	sf40	fd25	fd40
ASTRAL filtering	95%	40%	25%	40%
Number of Sequences	2115	1119	1294	1651
Number of Folds	25	25	25	27
Number of Superfamilies	47	37	137	158
Avg. Pairwise Similarity	12.8%	11.5%	11.6%	11.4
Avg. Max. Similarity	63.5%	33.9%	32.2%	34.3
Avg. Pairwise Similarity (within folds)	25.6%	17.9%	16.7%	17.4
Avg. Pairwise Similarity (outside folds)	10.4%	11.03%	11.2%	11.0

The percent similarity between two sequences is computed by aligning the pair of sequences using SW-GSM with a gap opening of 5.0 and gap extension of 1.0. "Avg. Pairwise Similarity" is the average of all the pairwise percent identities, "Avg. Max. Similarity" is the average of the maximum pairwise percent identity for each sequence i.e, it measures the similarity to its most similar sequence. The "Avg. Pairwise Similarity (within folds)" and "Avg. Pairwise Similarity (outside folds)" is the average of the average pairwise percent sequence similarity within the same fold and outside the fold for a given sequence.

Datasets, sf95 and sf40 are designed to evaluate the performance of remote homology prediction and were derived by taking only the domains with less than 95% and 40% pairwise sequence identity according to Astral [42], respectively. This set of domains was further reduced by keeping only the domains belonging to folds that (i) contained at least three superfamilies and (ii) one of these superfamilies contained multiple families. For sf95, the resulting dataset contained 2115 domains organized in 25 folds and 47 superfamilies, whereas for sf40, the resulting dataset contained 1119 domains organized in 25 folds and 37 superfamilies.

Datasets, fd25 and fd40 were designed to evaluate the performance of fold recognition and were derived by taking only the domains with less than 25% and 40% pairwise sequence identity, respectively. This set of domains was further reduced by keeping only the domains belonging to folds that (i) contained at least three superfamilies and (ii) at least three of these superfamilies contained more than three domains. For fd25, the resulting dataset contained 1294 domains organized in 25 folds and 137 superfamilies, whereas for fd40, the resulting dataset contained 1651 domains organized in 27 folds and 158 superfamilies.

### Binary Classifiers

The various one-versus-rest binary classifiers were constructed using SVMs. These classifiers used the recently developed [15] Smith-Waterman based profile kernel function (SW-PSSM), that has been shown to achieve the best reported results for remote homology prediction and fold recognition.

The SW-PSSM kernel computes a local alignment between two protein sequences, in which the similarity between two sequence positions is determined using a PICASSO like scoring function [43,44], and a position independent affine gap modeling scheme. The parameters for the affine gap model (i.e., gap-opening (*go*) and gap-extension (*ge*) costs), and zero-shift (*zs*) for our base classifiers were set to *go *= 3.0, *ge *= 0.75 and *zs *= 1.5. These values were determined in [15] by performing a large parameter study, and selecting the values that achieved the best binary classification performance. The binary base classifiers were trained using the widely used SVM^*light *^[45] program.

### Direct *K*-way Classifier

The direct *K*-way classification models were built using the publicly available implementation of the algorithm from the authors [31].

To ensure that the schemes are compared fairly, we use the same SW-PSSM kernel function used by the binary SVM classifiers. We tested the direct *K*-way classifiers using linear kernel functions as well, but the performance of the SW-PSSM kernels were substantially better.

### Performance Assessment Measures

The performance of the classification algorithms was assessed using the zero-one (ZE) and the balanced error rate (BE) [19]. The zero-one error rate treats the various classes equally and penalizes each misclassification by one. The balanced error rate accounts for classes of varying size and assigns a lower penalty for misclassifying a test instance belonging to a larger class. The motivation behind balanced error is that larger classes are easier to predict just by chance alone and it rewards a classifier if it can also correctly predict test instances belonging to smaller classes. Following the common practice [19], we set the error of each misclassification to be inversely proportional to its true class size.

In addition, the performance of the various classifiers was evaluated using the previously established approach for evaluating fold recognition methods introduced in [46,47] that does not penalize for certain types of misclassifications. For each test instance, this scheme ranks the various classes from the most to the least likely and a test instance is considered to be correctly classified if its true class is among the highest-ranked *n *classes (i.e., top_*n*_). The classes in the ranked list that are within the same next higher-level SCOP ancestral class are ignored and do not count towards determining the highest-ranked *n *classes. That is, in the case of fold recognition, the folds that are part of the same SCOP class as the test instance are ignored and they do not count in determining the *n *highest-ranked predictions. Similarly, in case of remote homology detection, this scheme ignores the superfamilies that are part of the same SCOP fold as the test sequence. Using a small value for *n *that is greater than one, this measure assesses the ability of a classifier to find the correct class among its highest ranked predictions, and by penalizing only for the substantially wrong mispredictions (i.e., different SCOP classes or folds), it can assess the severity of the misclassifications of the different schemes. In our experiments we computed the error rates for *n *= 1 and *n *= 3.

### Training Methodology

For each dataset we separated the proteins into test and training sets, ensuring that the test set is never used during any parts of the learning phase.

For sf95 and sf40 (fd25 and fd40), the test set is constructed by selecting from each superfamily (fold) all the sequences that are part of one family (superfamily). Thus during training, the dataset does not contain any sequences that are homologous (remote homologous) to the sequences in the test set and thus allows us to evaluate/assess remote homology prediction (fold recognition) performance. This is a standard protocol for evaluating remote homology detection and fold recognition and has been used in a number of earlier studies [13-15,48].

The models for the two-level approach can be learned in three phases by first splitting the training set into two sets, one for learning the first-level model and the other for learning the second-level model. In the first phase, the *k *one-vs-rest binary classifiers are trained using the training set for the first level. In the second phase, each of these *k *classifiers are used to predict the training set for the second level. Finally, in the third phase, the second-level model is trained using these predictions. However, due to the limited size of the available training set, we followed a different approach that does not require us to split the training set into two sets. This approach was motivated by the cross-validation methodology and is similar to that used in [19]. This approach first partitions the entire training set into ten equal-size parts. Each part is then being predicted using the *k *binary classifiers that were trained on the remaining nine parts. At the end of this process, each training instance has been predicted by a set of *k *binary classifiers, and these prediction outputs serve as training samples for the second-level learning (using the ranking perceptron or the structured SVM algorithm). Having learned the second-level model using the prediction values obtained from the first-level classifiers, we take the entire training set as a whole and retrain the first-level models. During the evaluation stage, we compute the prediction for our untouched test dataset in two steps. In the first step, we compute the prediction values from the first level model, which are used as features to obtain the final prediction values from the second level model. These predictions are then evaluated using the zero-one and the balanced error.

### Model Selection

The performance of the SVM depends on the parameter that controls the trade-off between the margin and the misclassification cost ("C" parameter in SVM-Struct), whereas the performance of ranking perceptron depends on the parameter *β *in Algorithm 1.

We perform a model selection or parameter selection step. To perform this exercise fairly, we split our test set into two equal halves of similar distributions, namely sets A and B. Using set A, we vary the controlling parameters and select the best performing model for set A. We use this selected model and compute the accuracy for set B. We repeat the above steps by switching the roles of A and B. The final accuracy results are the average of the two runs. While using the SVM-Struct program we let C take values from the set {0.0001,0.001, 0.005, 0.01, 0.02, 0.05, 0.1, 0.2, 0.5, 1.0, 2.0, 4.0, 8.0,10.0, 16.0, 32.0, 64.0, 128.0}. While using the perceptron algorithm we let the margin *β *take values in the set {0.0001,0.005, 0.001, 0.05, 0.01, 0.02, 0.5, 0.1, 1.0, 2.0, 5.0, 10.0}.

## Availability and Requirements

Additional data available at the website: 

## Authors' contributions

HR and GK designed the methods, and experimental setup. HR carried out the implementation of the various methods, and computational experiments. HR wrote the manuscript under GK's technical supervision and mentorship. Both authors read and approved the final manuscript.
